# Differential Dependencies of Monocytes and Neutrophils on Dectin-1, Dectin-2 and Complement for the Recognition of Fungal Particles in Inflammation

**DOI:** 10.1371/journal.pone.0045781

**Published:** 2012-09-26

**Authors:** Jacqueline U. McDonald, Marcela Rosas, Gordon D. Brown, Simon A. Jones, Philip R. Taylor

**Affiliations:** 1 Institute of Infection and Immunity, Cardiff University School of Medicine, Cardiff, United Kingdom; 2 Section of Infection and Immunity, Institute of Medical Sciences, University of Aberdeen, Aberdeen, United Kingdom; University of São Paulo, Brazil

## Abstract

We have re-investigated the role of the complement system and the non-opsonic pattern recognition receptors dectin-1 and dectin-2 in the recognition of fungal particles by inflammatory neutrophils, monocytes and macrophages. We have used *in vivo* and *ex vivo* models to study the recognition and response of these cells: i) We confirm previous observations regarding the importance of complement to neutrophil but not monocytic responses; ii) We show that dectin-1 is important for driving inflammatory cell recruitment to fungal stimuli and that it biases the immediate inflammatory response to one that favors neutrophil over monocyte recruitment; iii) We show that dectin-2 contributes to the physical recognition of fungal particles by inflammatory monocytes/macrophages, but is also expressed on neutrophils, where we show it has the potential to contribute to cellular activation; iv) Additionally, we show that serum-opsonization has the potential to interfere with non-opsonic recognition of fungal particles by dectin-1 and dectin-2, presumably through masking of ligands. Collectively these roles are consistent with previously described roles of dectin-1 and dectin-2 in driving inflammatory and adaptive immune responses and complement in containing fungal burdens. This study emphasizes the importance of heterogeneity of receptor expression across myeloid cell subsets in protective immune responses.

## Introduction

Neutrophils often only interact significantly with pathogens, such as fungi, after opsonization [1], whereas monocytes do not require opsonization [2]. Our studies of dectin-1 [3], [4], [5] demonstrated an additional mechanism for fungal recognition by neutrophils [5]. Both complement and dectin-1 can be considered ‘*primary pathogen recognition systems*’ in that they mediate the direct interaction of pathogens with immune cells and are fundamental to cellular processes such as phagocytosis. Without these systems, responses of other pattern recognition receptors (e.g. Toll-like receptors) are often impaired. In the mouse, dectin-1 seems to play a role in the recognition of non-opsonized *Candida albicans* by inflammatory cells and macrophages (MØ)^2^, but appeared redundant in the killing of opsonized yeast [5].

*C. albicans* is one of the main causes of mycoses worldwide. As with many fungal species, the cell wall is carbohydrate rich and a plethora of potential immune receptors may contribute to the immune recognition process [6]. We showed that complement C3 is important for the control of initial *C. albicans* burdens, but complement seems dispensable for the induction of inflammatory responses as C3-deficient mice exhibited enhanced inflammatory mediator production during infection [7]. Dectin-1-deficiency is associated with increased susceptibility to infection with *C. albicans* [5], perhaps mostly via oral/gastrointestinal routes [8], and as mentioned above this is associated with defective MØ recognition of *C. albicans* and impaired initiation of anti-fungal inflammatory responses [5], [8]. The importance of dectin-1 in antifungal responses is now highlighted in several experimental fungal infection models [5], [9], [10], and the increased susceptibility of dectin-1-deficient mice to *C. albicans* infections is supported by the discovery of dectin-1-deficient humans with recurrent mucocutaneous candidiasis [11], [12]. Notably, dectin-1-deficient mice infected with *C. albicans* elicit normal adaptive immune responses as illustrated by IFNγ and IL-17 production by splenocytes [13]. Dectin-2 is another lectin primarily expressed by MØ and dendritic cells (DC) and has specificity for ‘high-mannose’-like structures [14], [15], [16]. Dectin-2 uses the FcεRγ-chain [17], [18], [19] to signal via Syk, Card9 and MAPK dependent pathways, just like dectin-1 [13], [20], [21], [22]. Dectin-2 is important for the generation of Th17-like adaptive immune response, and together with dectin-1 coordinates Th1-like responses [18]. The involvement of dectin-2 in Th17 development has subsequently been confirmed in dectin-2-deficient mice, which display increased susceptibility to infection, similar to that observed under IL17-deficiency [23]. However, the susceptibility of these individual lectin-deficient mice to *C. albicans* infection is relatively mild when compared to that presented with Card9-deficiency, the common adapter signaling molecule for these lectins [21]. This likely reflects the redundancies of these receptors in infectious models, but also the multiple other receptors impacted by Card9 deficiency [21], [24].

Thus, experimental data indicate a role for both complement and dectin-1 in the recognition of fungi by neutrophils. Additional roles for dectin-1 and for dectin-2 have been identified in the generation of the adaptive immune response. It is logical that the primary role of complement and dectin-1 in controlling fungal numbers is mediated at least in part through neutrophils, a principal cell of host defense in fungal infection. The relatively mild impact of dectin-1-deficiency compared to C3-deficiency on the growth of *C.albicans in vivo* [5], [7] is indicative of the redundancy of innate immune recognition systems *in vivo*, but as other data from our laboratories has indicated, the study of gene-targeted animals does not always reveal the role of a defined protein or system in an immune response [13], [18], [25].

In this study, we have reassessed the role of dectin-1, dectin-2 and complement in fungal recognition and fungal-driven inflammation using *in vivo* and ex vivo analysis to determine the contribution of these mechanisms to the host defense process. We selected these effectors, as opposed to, for example, the macrophage mannose receptor which appears to have a secondary role in fungal recognition [26], as our study was focused on the primary recognition events. We have characterized a higher-dose model of zymosan peritonitis with the aim of establishing the role of dectin-1 in the inflammatory process and being able to examine the mechanisms used by inflammatory cells to respond to the challenge. Zymosan peritonitis was chosen because it is self-resolving and has been extensively used in the analysis of other molecular systems. We provide evidence for a significant level of interdependence between these receptor systems based on cellular heterogeneity.

## Materials and Methods

### General Reagents

OPTI-Modified Eagle’s Medium (OPTI-MEM), Fetal Calf Serum (FCS) and Alexa-fluor405 and Pacific Orange labeling kits were all obtained from Invitrogen. Fluorescein isothiocyanate isomer I (FITC) was purchased from Sigma. The Phycolink Peridinin-chlorophyll-protein complex (PerCP)-labeling kit was obtained from Europa Bioproducts. The Fugene 6 transfection reagent was obtained from Roche. Microscopy Hemacolour cell stain was from Merck. Flow-cytometric counting beads were from AbD Serotec. Unless otherwise stated, all generic reagents were obtained from Sigma or Invitrogen. All reagents were used according to manufacturer’s directions unless specified otherwise.

### Mice, Primary Cells and Peritonitis

129S6/SvEv and 129S6/SvEv.*Clec7a* ^−/−^ [5] mice were obtained from our own colonies. To induce sterile peritonitis, mice were injected intraperitoneally with 1 ml 2% (w/v) BIOgel polyacrylamide beads (BIO-Rad) or 2×10^7^ zymosan (Molecular Probes) particles in 100 µl of PBS usually up to 7 days prior to peritoneal lavage. Inflammatory cells were collected after sacrifice by peritoneal lavage with ice-cold 5 mM EDTA in PBS. Resident peritoneal cells were similarly collected from naive animals. To remove BIOgel particles from cell preparations the cells were passed through 40 µm cell strainers (BD).

### Fluorescent Labelling of Zymosan and *C. albicans*

Zymosan (Invitrogen) was labeled with either Alexa-Fluor405 (A405)-succinimidyl ester (Invitrogen) or FITC (Sigma) and live *C.albicans* SC5314 was labeled with Pacific Orange succinimidyl ester (Invitrogen) as previously described [27]. The labelled Zymosan/*C.albicans* was then washed (350×*g*, 5 minutes) four times with excess PBS or medium and resuspended in assay medium for ready for use, including opsonization. *C.albicans* was grown and serum opsonized as previously described [5]. Apart from titrations indicated in the text, serum opsonisation was achieved with 50% (v/v) mouse serum.

### Flow-cytometry

Staining of cells for flow cytometry was performed according to standard protocols at 4°C in the presence of 2 mM NaN_3_ as previously described [4], [25]. For intracellular TNF assays, resident peritoneal cells were washed and plated in 96-well v-bottomed plates (3×10^5^ cells/well) in 100 µl of RPMI1640, 10% (v/v) FCS, 50 U/ml penicillin, 50 µg/ml streptomycin, 2 mM L-glutamine containing GolgiPlug (BD). Zymosan-Alexa Fluor 405 was added (10 µg in 100 µl). Plates were incubated for 3 hours at 37°C in a 5% CO_2_ incubator. The cells were resuspended and immediately fixed with 1% formaldehyde in PBS for 20 min at 4°C. Cells were permeabilized for staining by including 0.5% saponin (Sigma-Aldrich) in all blocking and washing buffers. Antibody staining was performed according to the standard protocols with this modification.

The following antibodies were used in this study: Ly-6G-PE/PE-Cy7 (clone 1A8; BD); Ly-6B.2-PE/APC/FITC (clone 7/4; AbD Serotec [28]); F4/80-PE/APC/Alexa Fluor 405 (clone Cl: A3-1; AbD Serotec); Anti-dectin-2 (D2.11E4 [16]); anti-CD11b-FITC (clone 5C6; AbD Serotec); anti-CD11b-APC or –APC-Cy7 (clone M1/70; BD biosciences); anti-dectin-1-Alexa Flour 647 (clone 2A11; Serotec); anti-TNF-APC (clone MP6-XT22; BD); multiple-species adsorbed anti-rat IgG-PE (Jackson Immunochemicals); Isotype control were either produced in house, or obtained from either BD or AbD Serotec. When secondary anti-rat antibodies were used in mutli-colour flow-cytometry, samples were subsequently blocked with 10% rat serum before staining with additional rat antibodies.

Samples were acquired on a CyAn ADP Analyser (3 laser) (Beckman-Coulter) and data was analyzed using Summit (Beckman-Coulter) or FlowJo (Treestar) software. Myeloid cell subsets were identified as previously described [28], [29]. In brief, Neutrophils will identified as Ly-6G^+^Ly-6B.2^+^, eosinophils were identified as F4/80^+^SSC^high^ [29], tissue resident MØ were identified by a characteristic F4/80^high^CD11b^high^ phenotype [29], [30]. In select experiments, only Ly-6G was used to identify neutrophils (as indicated). At the 4 hour time point after zymosan administration, Ly-6C^+^ monocytes were identified by their Ly-6B.2^+^Ly-6G^–^F4/80^low^ phenotype [28]. Fourteen to 18 hours after inflammatory stimuli, early inflammation-associated monocytes and MØ were identified after exclusion of eosinophils and residual tissue resident MØ, by their Ly-6G^–^Ly-6B.2^high/+^ phenotype [28], [29], [31]. The above mentioned-gating strategies do not incorporate the relatively low number of ‘DC-like’ cells, which have a CD11b^+^F4/80^+^ phenotype [32]. During the time course analyses, “Total Monocyte/MØ” were defined as F4/80^low-high^CD11b^+/high^ cells (after exclusion of eosinphils), a strategy that would have included the relatively low number of ‘DC-like’ cells.

### Retroviral Transduction of NIH3T3

NIH3T3 were retrovirally transduced using retroviral vectors that had been packaged in phoenix cells as described in detail elsewhere [33]. The retroviral vectors pMXs-IP:Fcer1g [18] and the empty vector controls, pFBneo (Stratagene), pMXs-IP [34] and pMXs-IZ [25], [35], have been previously described. pMXs-IZ:dectin-2 was generated by amplifying the dectin-2 coding sequence (alpha isoform) by PCR using pFBneo:dectin-2-HA [16] as a template and the specific primers: 5′-AAAGGAATTCCACCATGGTGCAGGAAAGACAATC-3′ 5′-AAGGAATTCATAGGTAAATCTTCTTCATTTC-3′. The PCR product was digested and cloned into the pMXs-IZ vector using the *Eco* RI restriction enzyme and sequence fidelity was confirmed by sequencing (Central Biotechnology Services, Cardiff University School of Medicine). After retroviral transduction of the cell lines they were selected in antibiotics and subsequently maintained under selection (Zeocin, Invitrogen): 400 µg/ml; Puromycin (Sigma): 3 µg/ml). Expression of dectin-2, by the transduced cells was confirmed by flow-cytometric analysis [16]. The retroviral vector pFBneo:dectin-1-HA has also been reported previously [36] and was used in conjunction with pFBneo to generate new cell lines for this study in exactly the same way as described above except using G418 (Invitrogen) at 400 µg/ml for selection of transductants.

### *Ex vivo* and *in vitro* Cell:Microbe Interaction and Activation Assays

For the study of inflammatory cells, peritoneal cells were harvested 16–18 hours after BIOgel injection. Cells were washed with OptiMem, 10% (v/v) FCS, 50 units/ml penicillin, 50 µg/ml streptomycin, 2 mM L-glutamine, 30 µM β-mercaptoethanol), counted and then resuspended at 10^5^ cells per 60 µl of medium containing 3′-(p-aminophenyl) fluorescein (APF) (5 µM) in a v-bottomed 96 well plate. The cells were incubated at 37°C for 30 minutes to load the cells with APF. Next, 40 µl of stimulants (e.g. 2×10^6^ particles of zymosan or 2×10^6^ live C. albicans yeast) diluted in medium were added to each well and the plate was placed in a 37°C water bath for 15 minutes in a 5% CO_2_ incubator. During these incubations the plate was covered with a sterile gas permeable plate cover. The cells were then immediately put on ice. Cells were identified by immunostaining, using a quick stain protocol [25].

When using anti-dectin-2 (D2.11E4 [16]) or its rat IgG2a control (AbD Serotec) in blocking studies, the antibodies were included at 10 µg/ml in the APF preincubation step and the assays were otherwise conducted as described above.

For the analysis of the interaction of fluorescent zymosan with NIH3T3 transductants, cells were plated in 24-well plates at 0.5×10^4^ cells per well in DMEM supplemented with 10% FCS and penicillin/streptomycin. Next day, 10^8^ zymosan-FITC particles were resuspended in medium with various concentrations of mouse serum (0, 2, 10, 50 and 100%) and incubated at 37°C for 30 min with frequent agitation. After a 3 washes with medium, zymosan-FITC particles were resuspended and added to NIH3T3 transductants at 6×10^7^ particles per well in triplicates. Plates were incubated at 37°C and 4°C for 30 min and then, washed three times with ice-cold medium. Cells were kept on ice and scraped just before FACS acquisition on a CyAn ADP Analyzer (see above).

### Statistical Analyses

Statistical analysis was conducted using GraphPad Prism with the tests used indicated in the text where appropriate. Exact P values are displayed or abbreviated with the following convention: * = P<0.05; ** = P<0.01; *** = P<0.001.

## Results

### Characterisation of a ‘High-dose’ Zymosan Peritonitis Model

**Figure 1:**
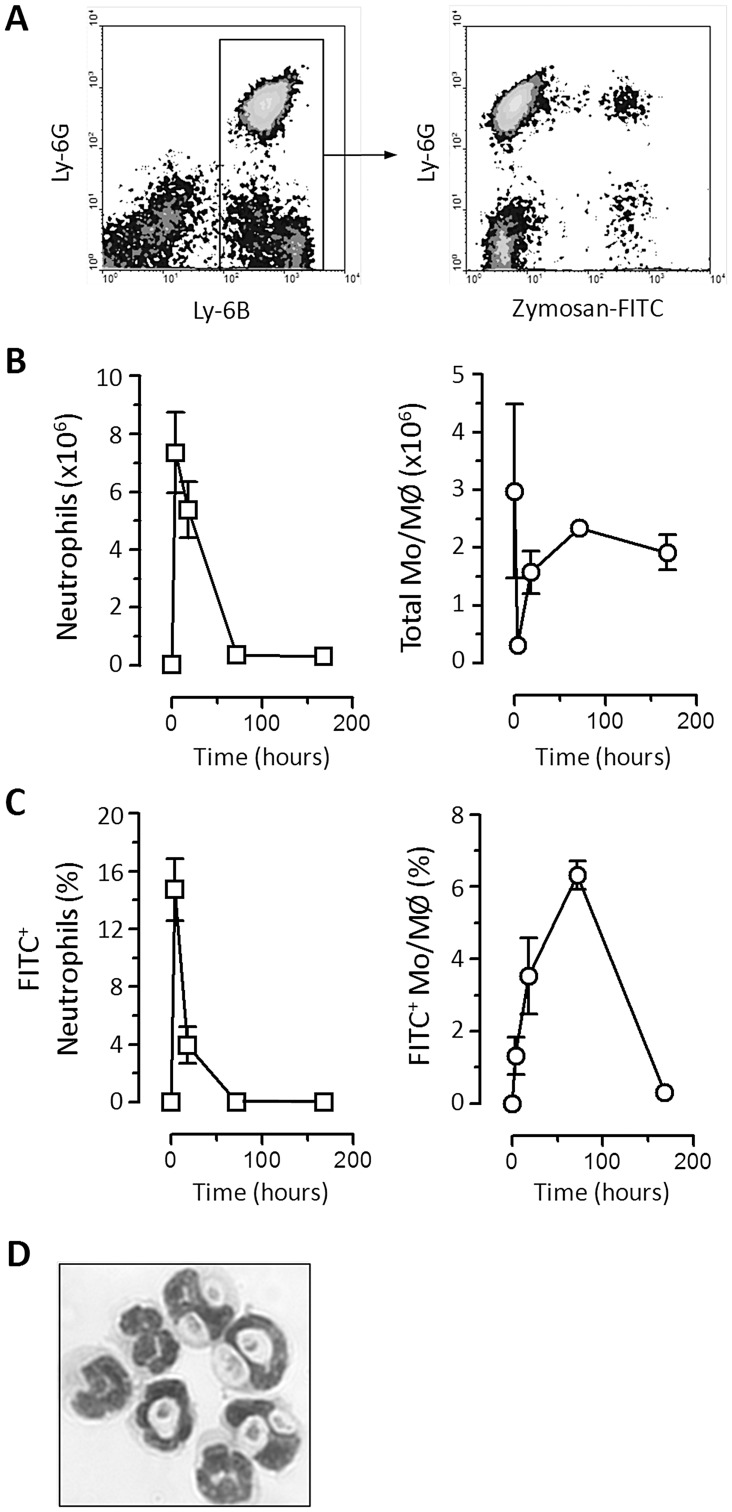
Characterization of high dose zymosan peritonitis. A) Representative flow-cytometric density plots showing the recruitment of inflammatory cells and the clearance of fluorescent (FITC)-labelled zymosan particles. The examples shown are taken from 18 hours after the administration of zymosan. Cells were identified as described in the methods. The panel on the left shows the gating of Ly-6B^+^ cells, which includes (right) Ly-6G^high^ neutrophils and Ly-6G^−^ monocytes and MØ. Both neutrophils and monocytes/MØ exhibit association with FITC-labeled zymosan at this time point (right panel). **B)** Graphical representation of the number of neutrophils (left) and all types of monocyte (Mo) and MØ combined (right) in the peritoneal cavity before and after acute zymosan peritonitis. n = 3 129S6/SvEv mice per group and is representative of 2 independent experiments. Data is shown as mean±SEM and cells were isolated from naïve animals (0 hours), and challenged mice 4, 18, 72 and 168 hours after induction of peritonitis. **C)** Graphical representation of the percentage of neutrophils and Mo/MØ present that are associated with zymosan, scored by flow-cytometry as indicated in (A) above. **D)** Photomicrograph, which is representative of neutrophils associated with zymosan in a 4 hour inflammatory infiltrate. Those neutrophils that are associated tend to have multiple particles, but only represent a minority of the total neutrophils present at this time (see (C) above).

First we examined the inflammatory kinetics of a single injection of 2×10^7^ FITC-labeled zymosan particles in wild-type (129S6/SvEv) mice. The fate of the zymosan particles was followed via the FITC-labeling as exemplified in **Figure 1A**. The inflammatory kinetic was typical of an acute self-resolving reaction with a rapid peak in neutrophil numbers, which was associated with a marked drop in the recoverable number of tissue resident MØ (**Figure 1B**). However monocyte/MØ numbers were subsequently restored by influx of new cells as inflammation proceeded (**Figure 1B**).

The neutrophils recruited during the early time points of the response exhibited most association with zymosan particles (**Figure 1C**). These zymosan-associated neutrophils often contained multiple particles (**Figure 1D**). The association of neutrophils and zymosan was already reduced by 18 hours post injection suggestive of a decline in available zymosan (**Figure 1C**). FITC-labelling of the newly recruited inflammatory monocytes and MØ was poor during these early time points with maximal association evident by day 3 (**Figure 1C**). Uptake of zymosan by tissue resident MØ is discussed in more detail below.

### *In vivo* Role of Dectin-1 in Zymosan Peritonitis and Inflammatory Cell:Zymosan Recognition

**Figure 2:**
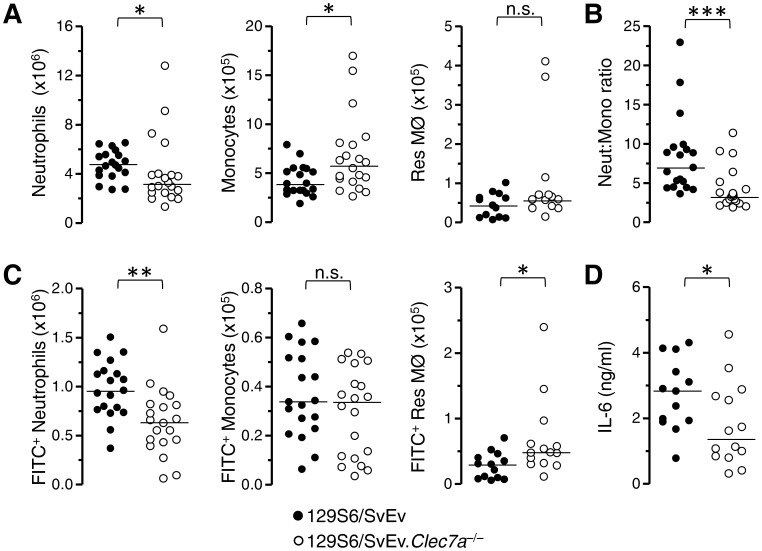
Characterization of high-dose zymosan peritonitis in Dectin-1-deficient mice. Wild type (closed circles) and dectin-1-deficient (*Clec7a* ^−/−^; open circles) 129S6/SvEv mice were injected intraperitoneally with 2×10^7^ zymosan particles and inflammatory infiltrates examined 4 hours later. **A)** Graphs showing the number of neutrophils, monocytes and residual tissue resident MØ (Res MØ) in the peritoneal cavity at this time. **B)** Graphical representation of a ratio of the absolute neutrophil and monocyte counts in each individual mouse. **C)** Graphs showing the numbers of those cells indicated in (A) above that are associated with FITC-zymosan. **D)** Quantification of IL-6 in the peritoneal lavage fluid of zymosan injected mice. For A,C and D data were normalized and pooled from 2 (Res MØ and IL-6 analysis) and 3 (Neutrophil and monocyte analysis) independent experiments. Each symbol represents an individual mouse. Horizontal lines denote medians. Data were analyzed by non-parametric Mann-Whitney U-test.

To determine the role of dectin-1 in the induction of inflammation in response to high doses of zymosan and the clearance of zymosan by neutrophils we examined the response of dectin-1-deficient (*Clec7a* ^−/−^) mice 4 hours after intraperitoneal administration of 2×10^7^ zymosan particles. Compared to the wild-type controls, the dectin-1-deficient mice exhibited a defect in neutrophil recruitment and an unexpected increase in monocyte recruitment (**Figure 2A**). A ratio of the absolute counts of neutrophils and monocytes demonstrated a clear bias in the recruitment of these subsets between both groups of mice (**Figure 2B**). The number of neutrophils associated with FITC-zymosan was also significantly lower in the absence of dectin-1 although the number of FITC-zymosan associated monocytes was not significantly altered (**Figure 2C**). Even though the number of recoverable tissue resident MØ were low (<5% of the numbers in naive mice) (**Figure 2A**), there were more dectin-1-deficient resident MØ associated with zymosan than wild type cells (**Figure 2C**). Since we have previously observed a robust dectin-1-dependent IL-6 response at this time point, we again assessed IL-6 production as a marker of cellular activation. The IL-6 present in the peritoneal cavity was significantly reduced in the dectin-1-deficient mice compared to the controls (**Figure 2D**).

### *Ex vivo* Characterisation of Dectin-1 and Complement Involvement in Fungal Recognition

**Figure 3:**
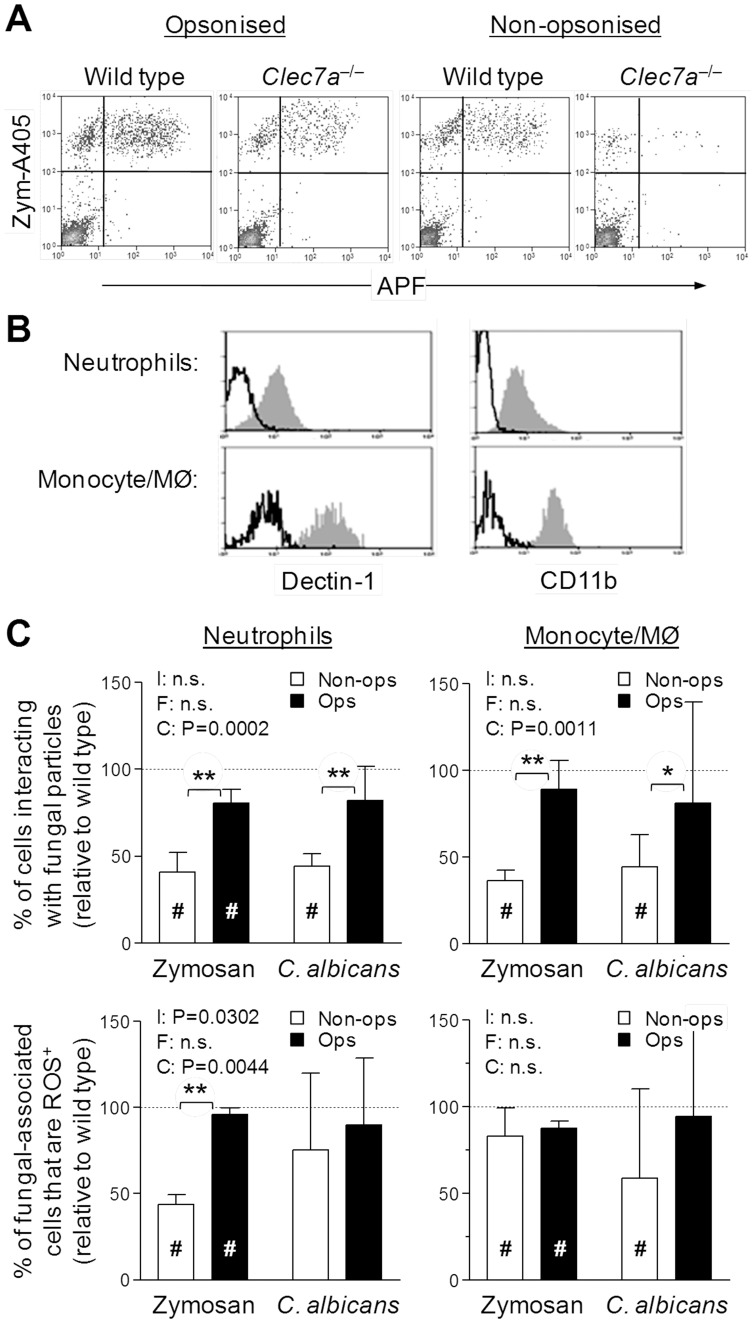
*Ex vivo* recognition of yeast particles and live fungi by inflammatory cells. A) Representative flow-cytometric analysis, gated on Ly-6G^+^ neutrophils, after coincubation of BIOgel-elicited inflammatory cells from wild type or dectin-1-deficient (*Clec7a* ^−/−^) 129S6/SvEv interaction with serum-opsonized or non-opsonized zymosan. Positive staining for the A405-labelled zymosan identifies the neutrophils that are associated zymosan and only these cells exhibit conversion of APF, the ROS reporter. **B)** Representsative flow-cytometric analysis of CD11b and dectin-1 expression by inflammatory neutrophils and monocyte/MØ. Data represents specific receptor staining (shaded histograms) and isotype control staining (bold lines). Data representative of 2 independent experiments and consistent with previous experiments with thioglycollate. **C)** Inflammatory cells were loaded with APF and then incubated with serum-opsonized or non-opsonized A405-labelled zymosan or Pacific Orange-labelled *C. albicans* for 15 minutes. After this time the association of the inflammatory cells with zymosan was measured by flow-cytometry (upper panels) and in those cells that were interacting with zymosan the evidence for fluorescent conversion of APF was also quantified (lower panels). Data is derived from three independent experiments and the data derived from the use of dectin-1-deficient cells is shown relative to wild type cells (100%) as mean±95% confidence interval (raw representative data from one of the 3 independent experiments are shown in the Figure S1). The impact of complement osponization (‘C’) and the use of different fungal particle used (‘F’) were assessed by Two-way ANOVA (‘I’  =  Interaction statistic). Samples in which the 95% confidence intervals do not overlap with the mean wildtype are specific indicated with a # symbol. Differences in impairment of response observed with dectin-1-deficient cells were further analysed by Bonferroni post-tests. P values derived from individual Bonferroni post-tests are indicated with bracketed pairs of samples.

The altered inflammatory process that occurred in the absence of dectin-1 makes the interpretation of the *in vivo* studies, with regards to the role of dectin-1 in neutrophil and monocyte recognition of zymosan difficult. To overcome this we examined the interaction of inflammatory neutrophils and monocyte/MØ *ex vivo*. Inflammatory myeloid cells were recovered from the peritoneal cavity after injection with BIOgel polyacrylamide beads. BIOgel was used because the inflammatory response that follows was not impaired by dectin-1-deficiency. The physical interaction between the inflammatory leukocytes and fluorescently-labeled zymosan (A405) or live *C. albicans* (Pacific Orange) was assessed by flow-cytometry after prior gating on neutrophils (Ly-6G^+^Ly-6B.2^+^) and monocyte/MØ (Ly-6G^–^Ly-6B.2^+^) [28], [29]. Examples of gated neutrophils are shown in **Figure 3A**. Conversion of APF by products of the respiratory burst to a fluorescent form only occurred in cells interacting with fungal particles (**Figure 3A**). Inflammatory neutrophils (Neut) and Monocyte/MØ (Mono/MØ), both expressed CD11b and dectin-1 as previously reported [4], but it was evident that inflammatory monocyte/MØ expressed more of both (**Figure 3B**).

Both, inflammatory neutrophils and monocyte/MØ from dectin-1-deficient mice exhibited a clear defect in the physical interaction with non-opsonized zymosan particles when compared to the wild-type mice (**Figure 3C**) (for specific data from a representative experiment see **supplemental** **Figure 1**). Recognition of non-opsonized zymosan by neutrophils was poor, but was more efficient by the inflammatory monocyte/MØ (**supplemental** **Figure 1**). When zymosan was opsonized with mouse serum, neutrophil recognition was substantially increased and dependence on dectin-1 for both cell types to interact with the zymosan particles was reduced (**Figure 3C**, upper panels). Of those cells that interacted with zymosan during the course of the experiment, a subset triggered respiratory burst as measured by APF conversion (**Figure 3C**, lower panels and **Figure S1**). Dectin-1-deficient neutrophils were substantially impaired in their ability to trigger this respiratory burst when non-opsonised zymosan was used, but exhibited little evidence of a defect when opsonised zymosan was used (**Figure 3C**, lower left panel). Dectin-1-deficient inflammatory monocyte/MØ only exhibited a minor defect in their ability to trigger a respiratory burst when interacting with zymosan (either opsonized or non-opsonized) (**Figure 3C**, lower right panel), but notably the presence or absence of opsonization had little effect on these cells.

The results obtained with live *C. albicans* were broadly consistent with those obtained with zymosan (**Figure 3C**). The main difference appeared to be a lower dependence on dectin-1 for triggering the respiratory burst in neutrophils in the absence of opsonization, once a physical interaction had occurred (**Figure 3C**, lower left panel). Similarly though, there was little dependence on serum opsonization for the respiratory burst of cells that had successfully interacted with the yeast particle/live *C. albicans*. (**Figure 3C**, lower right).

### Role of Dectin-2 in Inflammatory Cell Fungal Recognition

**Figure 4:**
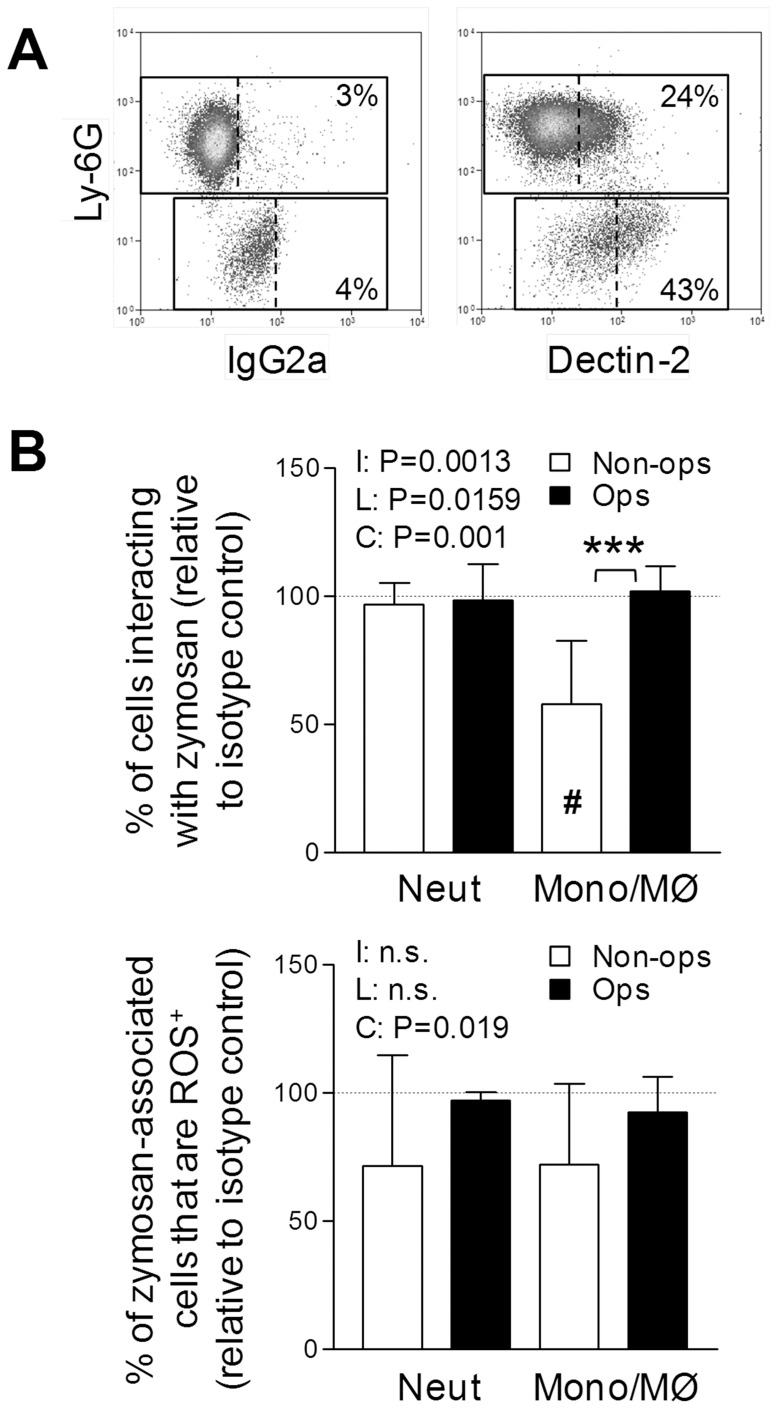
Assessment of dectin-2 function on inflammatory cells. A) Flow-cytometric plots showing expression of dectin-2 on the surface of inflammatory neutrophils (Ly-6G^+^) and monocyte/MØ (Ly-6G^−^). Data is pre-gated on Ly-6B.2^+^ cells and is representative of results obtained with pooled cells from 3 independent experiments. **B)** Assessment of the impact of blockade of dectin-2 (D2.11E4) on the recognition (upper graphs) and response to (lower graphs) zymosan of inflammatory neutrophils and monocyte/MØ from dectin-1-deficient mice. All bars show mean±95% confidence intervals of dectin-2-blocked cells relative to isotype control treated cells (100%, dotted line) from 3 independent experiments (raw representative data, which includes the isotype control data, from one of the 3 independent experiments are shown in Figure S2). Data were analysed by two-way ANOVA, two assess the differences caused by complement opsonization (‘C’) or the use of different cell lineages (‘L’). Samples in which the 95% confidence intervals do not overlap with the mean isotype control are specific indicated with a # symbol.

The other evident feature of the ability of the inflammatory cells to interact with the yeast particles was that dependency on dectin-1 was much less marked than has been observed with other inflammatory MØ populations, such as thioglycollate-elicited MØ [3], [4], [33]. Dectin-2 is transiently upregulated on inflammatory monocyte/MØ [16]. To investigate whether this could contribute to the alleviation of this dectin-1-dependency, we reassessed the expression of dectin-2 (**Figure 4A**) and following treatment with a blocking antibody (D2.11E4 [16], [18]) before performing recognition assays (**Figure 4B**). Low levels of dectin-2 surface expression were observed on neutrophils when sensitive staining protocols were adopted (**Figure 4A**). When anti-dectin-2 was employed (using dectin-1-deficient cells to help unmask its activity), we noted no effect on the recognition of zymosan by neutrophils (**Figure 4B** upper panel), but a consistent reduction in the recognition by inflammatory monocyte/MØ (**Figure 4B**, upper panel). Data from a representative experiment can be in **Figure S2**. For both cell types, the relative ability of the dectin-2-blocked dectin-1-deficient cells that had recognized zymosan to respond with ROS production was reduced when the zymosan was not opsonized. (**Figure 4B**, lower panel).

**Figure 5:**
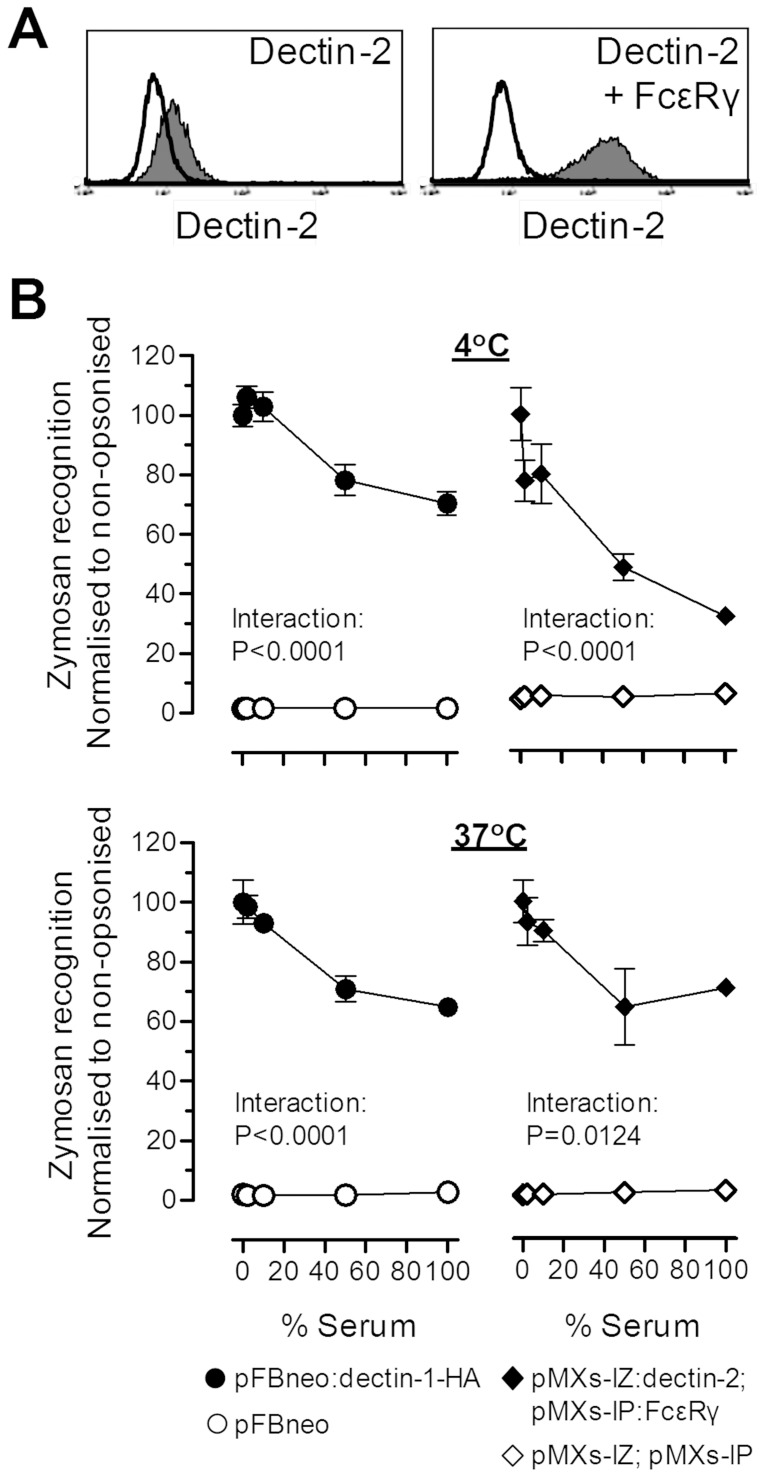
Inhibition of dectin-1 and -2-mediated fungal recognition by prior complement opsonization. **A)** Representative flow cytometric plots of dectin-2 surface expression on NIH3T3 retrovirally transduced with dectin-2 (left panel) and both dectin-2 and FcεRγ chain (right panel). **B)** Serum-opsonization inhibits the recognition of zymosan by both dectin-1 and dectin-2. NIH3T3 retrovirally transduced as indicated in the legend were incubated with zymosan that had been pre-incubated with serum at increasing concentrations and the interaction was monitored by flow cytometry. Data represents the mean±SEM of triplicates from a representative of 2 independent experiments. Data in (D) was analyzed by two-way ANOVA, with the significant Interaction statistics demonstrating the reduced binding of zymosan to the transduced cells after prior incubation with increasing concentrations of serum.

We also considered that prior opsonization of yeast particles with complement and other serum factors may in fact impair recognition of yeast particles, by non-opsonic recognition systems. For this purpose, we generated dectin-2 and FcεRγ-chain co-expressing NIH3T3 fibroblasts to compare to dectin-1 expressing NIH3T3 cells [36]. The heightened surface expression of dectin-2 in the presence of FcεRγ on these new cells was confirmed by flow cytometry (**Figure 5A**). Subsequently, the ability of dectin-1 and dectin-2-transduced NIH3T3 to recognize zymosan was studied after 30 min incubations at 4°C and 37°C with or without prior opsonization of the particles with increasing doses of serum (**Figure 5B**). Increasing concentrations of serum used in the opsonization of the zymosan particles resulted in reductions in the engagement of both alternative non-opsonic pathogen recognition systems.

## Discussion

Zymosan and other fungal particles have been extensively used *in vivo* to study acute inflammatory responses and the mechanisms that control them. As part of our previous studies on dectin-1 we examined the inflammatory responses to 10^5^−2×10^6^ live fungi or yeast particles [5]. Typically, zymosan has been used intraperitoneally in the 0.1–1 mg range for the study of peritonitis, which roughly equates to 2×10^7^−2×10^8^ particles, with some studies using as much as 10 mg (∼2×10^9^ particles). Increasing the number of particles quickly overwhelms the resident tissue phagocytes, invoking alternative inflammatory processes and leaving ‘excess’ zymosan in the cavity to be cleared by the recruited inflammatory cells. We have observed the impact of different dosing regimens on the mechanism of the acute inflammatory response when using more receptor-selective reagents and this may also represent an artificial reliance upon alternative inflammatory mechanisms in the absence of a primary receptor [25]. Our previous studies identified a role for dectin-1 in the activation of tissue resident phagocytes in response to fungal challenge, which is associated with the production of inflammatory mediators and the recruitment of inflammatory cells [5], [25]. We hypothesized that higher dosing regimens may have removed the need for dectin-1 in the induction of the inflammatory response, but it would also permit us to study the clearance of the residual fungal particles directly by the inflammatory neutrophils and monocytes.

Intraperitonal administration of 2×10^7^ zymosan particlesis associated with an acute inflammatory response characterized by disappearance of tissue resident MØ and influx of neutrophils and monocytes. Initially, a significant proportion of neutrophils were associated with multiple zymosan particles, but inflammatory monocyte numbers were low during this period and showed minimal interaction with zymosan particles. Peak zymosan association with inflammatory MØ was observed later in the inflammatory response after 3 days. Whilst only a small subset of these cells interacted with zymosan the cells tended to contain multiple particles. Given the kinetics of this interaction, it is conceivable that these MØ acquire zymosan through ingestion of apoptotic neutrophils or alternatively may represent the persistence of a small number of tissue resident MØ late in the inflammatory reaction [30], [37].

Studies in Dectin-1-deficient mice showed that dectin-1 was important for the recruitment of neutrophils in both high (2×10^7^ particles) and low dose [5] zymosan peritonitis. Interestingly, the dependency of inflammatory monocyte recruitment on dectin-1 in the low dose studies [5] was lost in these higher dose studies (this manuscript) indicating that alternate mechanisms promote the recruitment of monocytes in these circumstances and that dectin-1 is differentially important for neutrophil recruitment. This suggests that dectin-1 may bias towards a neutrophilic response, which could be assumed to favor fungal killing.

Additionally, these studies indicated that dectin-1 contributed to the recognition of fungal particles by *in vivo* elicited inflammatory neutrophils and monocytes. The number of neutrophils recognizing yeast particles was lower with dectin-1-deficiency even though there were less neutrophils and the number of inflammatory monocytes associated with zymosan was similar even thought there were more monocytes present. These data hint that dectin-1 was functionally active on both neutrophils and monocytes *in vivo*, however, the markedly altered inflammatory context makes confounds this conclusion so *ex vivo* studies were conducted to address the role of dectin-1 on these cells. We reproducibly observed a role for dectin-1 in the *ex vivo* recognition of and cellular responses of inflammatory neutrophils to fungal particles, which consistent with our previous study [5] and with expectations that complement is a fundamental mediator of neutrophil recognition, was more evident when the particle was non-opsonized.

The high dose of zymosan was associated with substantial disappearance of tissue resident MØ in both strains. In spite of this, the defective induction of IL-6 indicates that cellular activation was still impaired. Lastly, the residual tissue MØ from dectin-1-deficient mice that were associated with zymosan were significantly increased. However, normally the peritoneal cavity of a mouse has approximately 2−4×10^6^ tissue resident MØ, many of these are not recoverable during inflammation. Thus, since only a minority of tissue resident MØ are recoverable, the proposal that increased association between tissue resident MØ and fungal particles in the absence of dectin-1 could be a consequence of impaired clearance by inflammatory cells has the caveat that it could be subject to a selective bias of assaying a small number of residual cells. As expected, all of these observations are consistent with multiple mechanisms underlying the inflammatory response [5], [38].

During the course of these studies, we observed low dectin-2 expression on the surface of neutrophils. In the absence of complement, anti-dectin-2 slightly impacted on the response of inflammatory dectin-1-deficient neutrophils to zymosan. However, it did not affect the physical recognition process of neutrophils, which in the absence of complement and dectin-1 was extremely poor, consistent with this low surface expression of dectin-2 by neutrophils. Interestingly, Li et al. recently reported the role of dectin-1 in the activation of CD11b [39], our results are more consistent with a minimal role for dectin-1 in neutrophil activation by fungal particles, but this co-operative interaction could explain the more pronounced role we have observed for dectin-1 in the response to non-opsonised rather than opsonised fungal particles. It seems likely that *in vivo* other factors would contribute to CD11b activation, and it would be interesting in the light of these studies to determine if dectin-2 and other lectins could substitute for dectin-1.

Dectin-1-deficiency impaired the recognition of fungal particles by inflammatory monocyte/MØ *ex vivo*. These inflammatory monocytes exhibit an upregulation of dectin-2 expression [16] and both bone marrow cultures in GM-CSF (suggested to be similar to inflammatory monocyte derived cells [40]) and a cell line model that resemble these cultures identify dectin-2 as a second receptor for fungi alongside dectin-1 [18], [35]. Blockade of dectin-2 in dectin-1-deficient inflammatory monocyte/MØ further reduced, although not completely, their ability to elicit zymosan-mediated responses. Mincle [41], [42] is another likely candidate that could mediate this interaction, and like dectin-2 we have observed transient upregulation of the mRNA encoding mincle on inflammatory monocytes (data not shown). The fundamental role played by surface lectins in the recognition of fungal particles by inflammatory and tissue resident monocytic cells in conjunction with the observed impact of select deficiencies on the adaptive immune responses [5], [13], [18], [23] raises questions about the regulation of these events. Thus, the dependence on one receptor over another, for example in driving Th17 responses [13], [18], could be governed by: i) selective expression of these receptors by responding antigen presenting cell; ii) a selective display of ligands by the pathogen; or iii) by subtle differences in the signaling responses of these receptors.

Lastly, we considered that ‘excess’ *in vitro* complement opsonization may compete with non-opsonic receptors (as a direct competitive inhibitor). Prior *in vitro* opsonization of zymosan with high concentrations of serum directly inhibited its ability to interact with dectin-1 or dectin-2 transduced fibroblasts confirming this as a possibility. Our previous studies of dectin-1 ‘specific’ agents indicated that the converse also occurs, with rapid clearance of a limited challenge by mononuclear phagocytes restricting alternative inflammatory mechanisms [25], speculated to be something like complement.

Collectively, these studies are consistent with the host response to infection being reliant on the cellular heterogeneity and tissue microenvironment at the site of infection and, perhaps critically, the display of ligands by distinct morphologies of a pathogen and the number of infecting organisms.

## Supporting Information

Figure S1 ***Ex vivo*** **recognition of yeast particles and live fungi by inflammatory cells. A)** Inflammatory cells were loaded with APF and then incubated with serum-opsonized or non-opsonized zymosan for 15 minutes. After this time the association of the inflammatory cells with zymosan was measured by flow-cytometry (upper panels) and in those cells that were interacting with zymosan the evidence for fluorescent conversion of APF was also quantified (lower panels). **D)** The experiments shown in (C) were repeated with live pacific orange-labelled *C. albicans*. Both (C) and (D) are representative experiments from 3 independent experiments and data shown represented the mean±SEM of triplicates.
(TIF)

Figure S2 **Assessment of dectin-2 function on inflammatory cells.** Assessment of the impact of blockade of dectin-2 (D2.11E4) on the recognition (upper graphs) and response to (lower graphs) zymosan of inflammatory neutrophils and monocyte/MØ. Data represents mean±SEM of triplicates from a representative of 3 independent experiments.
(TIF)
