# Supplementary figures and images for: Differential Dependencies of Monocytes and Neutrophils on Dectin-1, Dectin-2 and Complement for the Recognition of Fungal Particles in Inflammation
Source: PLoS One. 2012 Sep 26;7(9):e45781. doi: 10.1371/journal.pone.0045781 (PMC3458947; doi:10.1371/journal.pone.0045781)

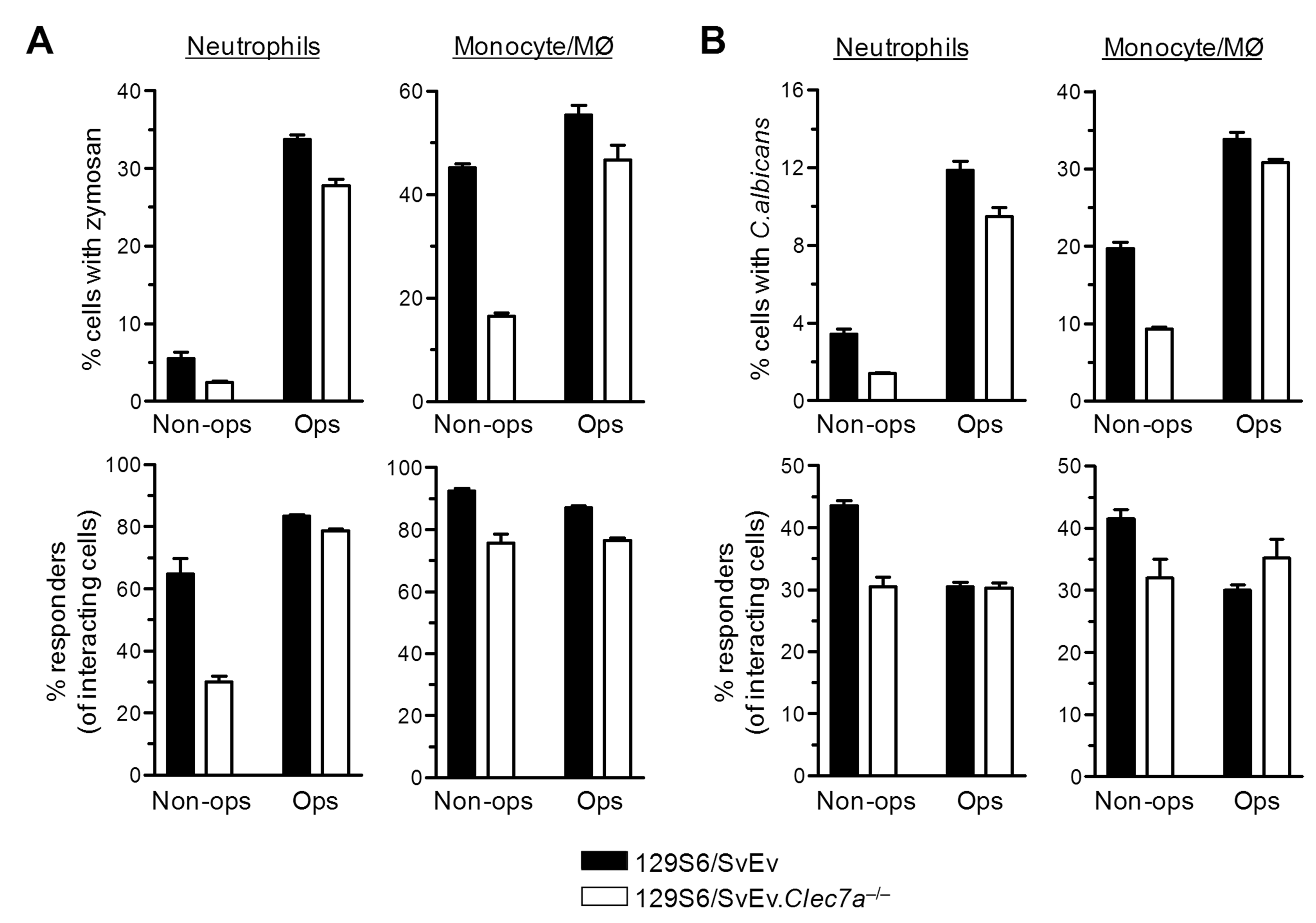

Supplement: Figure S1 — Ex vivo recognition of yeast particles and live fungi by inflammatory cells. A) Inflammatory cells were loaded with APF and then incubated with serum-opsonized or non-opsonized zymosan for 15 minutes. After this time the association of the inflammatory cells with zymosan was measured by flow-cytometry (upper panels) and in those cells that were interacting with zymosan the evidence for fluorescent conversion of APF was also quantified (lower panels). D) The experiments shown in (C) were repeated with live pacific orange-labelled C. albicans. Both (C) and (D) are representative experiments from 3 independent experiments and data shown represented the mean±SEM of triplicates. (TIF) [file pone.0045781.s001.tif]

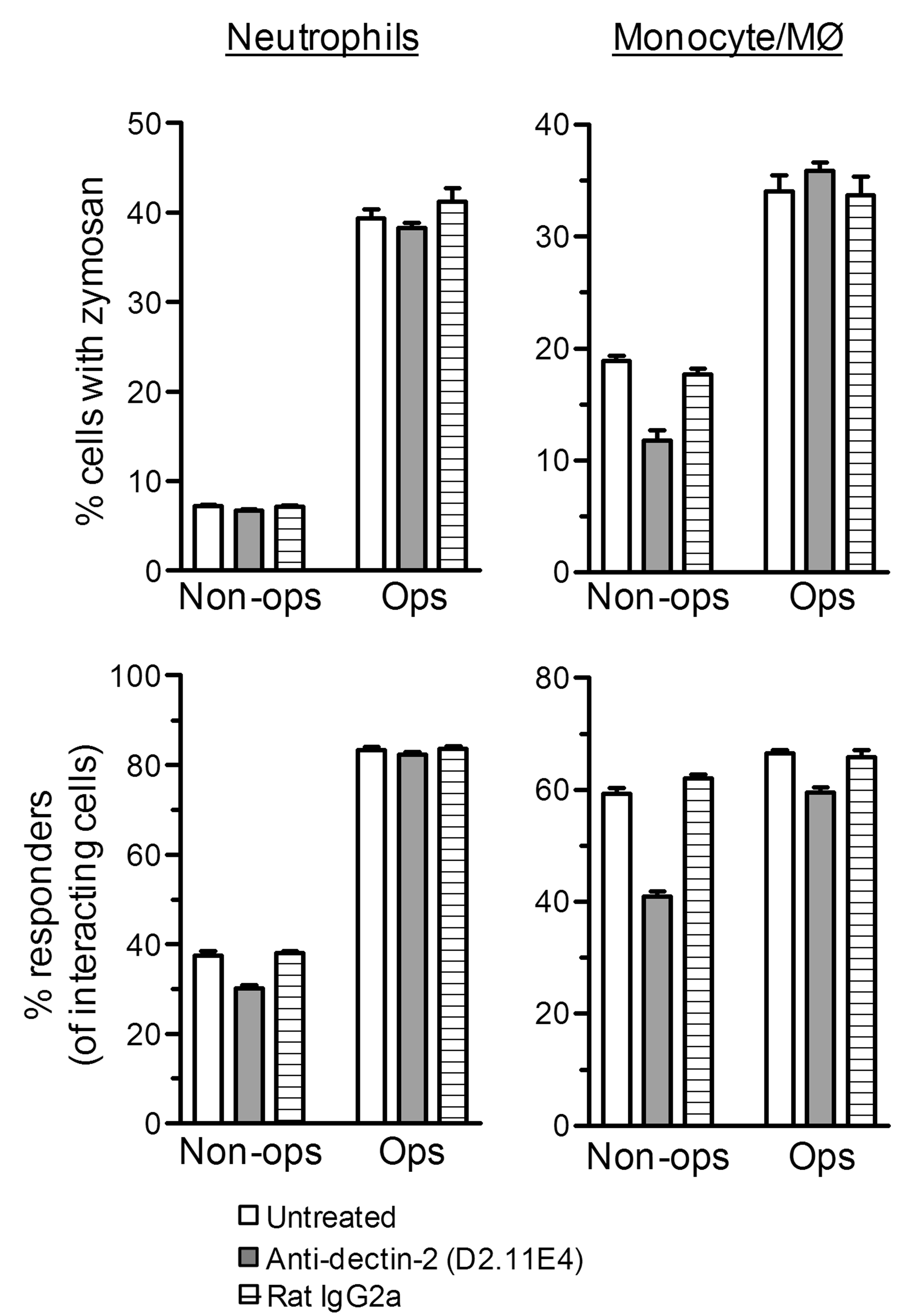

Supplement: Figure S2 — Assessment of dectin-2 function on inflammatory cells. Assessment of the impact of blockade of dectin-2 (D2.11E4) on the recognition (upper graphs) and response to (lower graphs) zymosan of inflammatory neutrophils and monocyte/MØ. Data represents mean±SEM of triplicates from a representative of 3 independent experiments. (TIF) [file pone.0045781.s002.tif]
